# Draft Genome Sequence of *Pseudomonas abietaniphila* KF701 (NBRC110664), a Polychlorinated Biphenyl-Degrading Bacterium Isolated from Biphenyl-Contaminated Soil

**DOI:** 10.1128/genomeA.00473-15

**Published:** 2015-05-14

**Authors:** Hidehiko Fujihara, Atsushi Yamazoe, Akira Hosoyama, Hikaru Suenaga, Nobutada Kimura, Jun Hirose, Takahito Watanabe, Taiki Futagami, Masatoshi Goto, Kensuke Furukawa

**Affiliations:** a Department of Food and Nutrition, Beppu University, Beppu, Japan; b Biological Resource Center, National Institute of Technology and Evaluation (NITE), Tokyo, Japan; c Bioproduction Research Institute, National Institute of Advanced Industrial Science and Technology (AIST), Tsukuba, Japan; d Faculty of Engineering, Department of Applied Chemistry, University of Miyazaki, Miyazaki, Japan; e Research Institute for Sustainable Humanosphere, Kyoto University, Kyoto, Japan; f Faculty of Agriculture, Education and Research Center for Fermentation Studies, Kagoshima University, Kagoshima, Japan; g Faculty of Agriculture, Laboratory of Future Creation Microbiology, Kyushu University, Fukuoka, Japan

## Abstract

*Pseudomonas abietaniphila* KF701 utilizes biphenyl as a sole source of carbon and degrades polychlorinated biphenyls (PCBs). Here, we report the 6,886,250-bp draft genome sequence of KF701, which contains 6,315 coding sequences and 59.4 mol% G+C content. The strain possesses genes for biphenyl catabolism and other genes that mediate the degradation of benzoate, salicylate, and phenol.

## GENOME ANNOUNCEMENT

Polychlorinated biphenyls (BCBs) have been recognized as serious environmental pollutants (1). Biphenyl-utilizing bacteria cometabolize certain PCB congeners into chlorobenzoic acids through oxidation by biphenyl-catabolic enzymes. The biphenyl catabolic *bph* genes were first cloned from *Pseudomonas pseudoalcaligenes* KF707 (2). Since then, a number of PCB-degrading bacteria have been identified, including both Gram-negative and Gram-positive bacteria (3). Some strains possess very similar, if not identical, *bph* genes, while others possess diversified *bph* genes (4). The purpose of this study was to explore how *bph* genes are organized, transferred, and rearranged by sequencing the genomes of various PCB degraders isolated from the same site. We isolated 14 PCB-degrading bacterial strains (KF strains), including *Pseudomonas abietaniphila* KF701 (formerly *Pseudomonas graminis* KF701) from biphenyl-contaminated soil in Kitakyushu, Japan (4).

Whole-genome shotgun sequencing of *P. abietaniphila* KF701 was performed by the National Institute of Technology and Evaluation (NITE) using a combination of shotgun sequencing on a 454 Roche GS FLX+ system (Roche) and paired-end sequencing on a HiSeq sequencing system (Illumina). The Newbler software package (version 2.6; Roche) was used for genome assembly. The draft genome was composed of 140 contigs (>537 bp) totaling 6,886,250 bases, with a G+C content of 59.4 mol%. The *N*_50_ contig size and the largest contig size were 90,844 bp and 392,029 bp, respectively.

Rapid genome annotation using the RAST annotation server (5) described 6,315 coding sequences (CDSs), 58 tRNA sequences, and 4 rRNA sequences. The coding sequences were classified into 547 subsystems, including cofactors, vitamins, prosthetic groups, and pigments (*n* = 328 CDSs), phages, prophages, transposable elements, and plasmids (*n* = 17 CDSs), motility and chemotaxis (*n* = 152 CDSs), metabolism of aromatic compounds (*n* = 146 CDSs), and metabolism of carbohydrates (*n* = 526 CDSs). A comparison of *P. abietaniphila* KF701 with other *Pseudomonas* strains within the RAST server database identified *Pseudomonas syringae* pv. syringae B728a (taxonomy ID, 205918.7) as its closest neighbor, with a score of 515, followed by *P. syringae* pv. phaseolicola 1448A (taxonomy ID, 264730.9), with a score of 481.

Functional annotations were compared with the Kyoto Encyclopedia of Genes and Genomes (KEGG) (6). Strain KF701 possessed *bph* genes very similar to those of *Pseudomonas putida* KF715 (7), benzoate-degrading genes via the hydroxylation pathway, and entire genes of the salicylate and phenol degradation pathways.

### Nucleotide sequence accession numbers. 

The draft genome sequence of *P. abietaniphila* KF701 has been deposited at DDBJ/EMBL/GenBank under the accession numbers BBQJ01000001 to BBQJ01000140.
